# Supplementary material for: Trading off fiscal budget adherence and child protection
Source: PLoS One. 2022 Mar 24;17(3):e0261664. doi: 10.1371/journal.pone.0261664 (PMC8947134; doi:10.1371/journal.pone.0261664)
Supplement: S1 Appendix — (DOCX) [file pone.0261664.s001.docx]

**S1 Appendix**

**Data**

The data set used in the paper combines Danish administrative register data with data on municipal level budgets and accounts. The rich Danish register data consists of longitudinal micro level data, which are accessible in anonymized form through Statistics Denmark’s facility for researchers at Danish research institutions. The registers include administrative information on a wide array of socioeconomic characteristics, including detailed information on social services to at-risk children and families including start and end date for each service. This allows us to construct a data set with care status for all children at the monthly level linked to socioeconomic characteristics of the child and parents. S1 Fig shows municipal averages of the number of children in out-of-home care for the period 2007 to 2016 by quartile.

**S1 Fig. The number of children in out-of-home care per municipality, 2007-2016.**


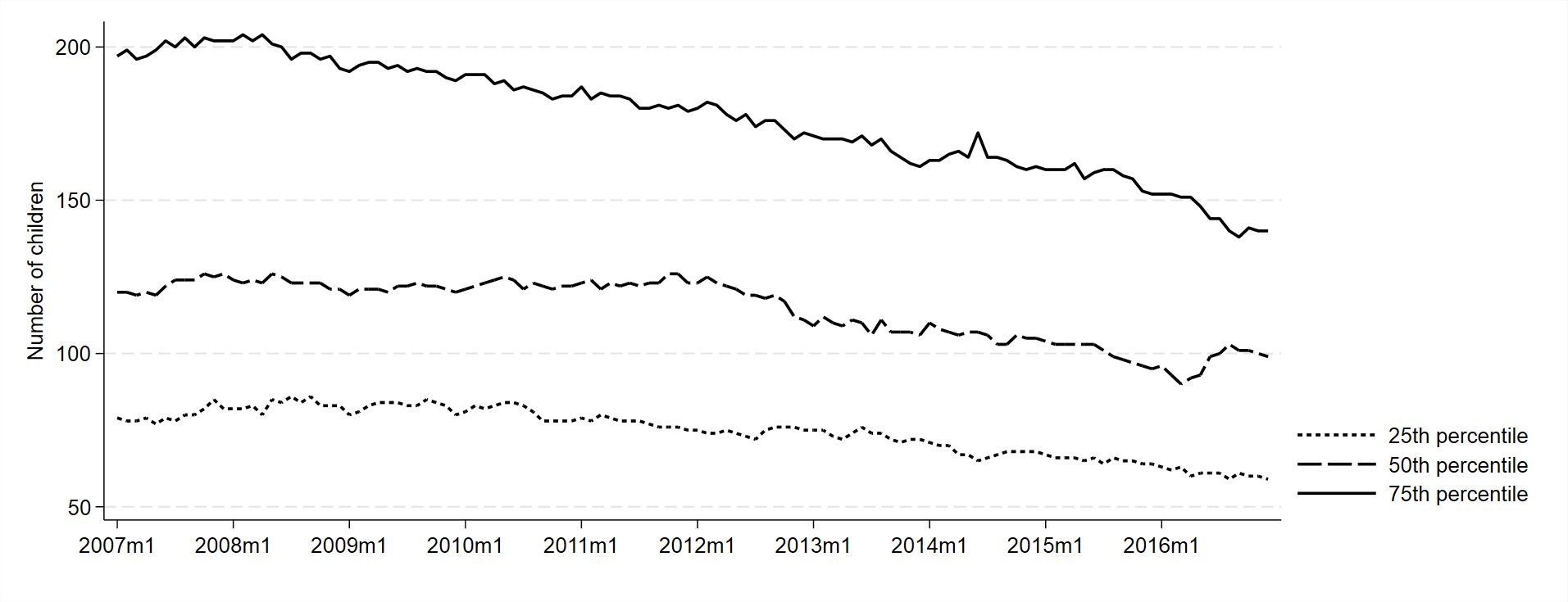


*Source:* Own calculation based on register data. Distribution of the number of children in care in municipalities in Denmark. The solid line is the 75^th^ percentile municipality with respect to number of children in care, dashed line is the median municipality, and the dotted line the 25^th^ percentile municipality. Monthly observations from 2007-2016.

While data on municipalities’ budgets and accounts are recorded annually, the individual register information allows us to calculate exactly how many children were in out-of-home care each month in the period. Furthermore, we can divide these “care months” according to the type of care. The cost of each type of care varies significantly, as shown in S1 Table, which shows average prices per care type.

**S1 Table. Annual average price by type of care, 2016.**

|  | Price per month (Euro) |
| --- | --- |
| Living with relatives | 1,570 |
| Foster families | 5,640 |
| Institutional care | 12,700 |
| Boarding schools | 4,250 |
| Own room, dorm or similar | 3,000 |
| Secured institutional care | 31,380 |
| Social educational residency | 12,160 |
| Ship project | 6,400 |

*Source*: Socialstyrelsen, SocialAnalyse nr. 2, 03.2017, Table 3. The prices are average prices across all municipalities. Prices exclude federal refunds.

By combining the information on average annual prices of different types of care shown in Table A1 with register based monthly information on each municipality’s interventions by care type, *h*, we calculated the total monthly expenses used on all types of care for each municipality, *k*:

|  | ${cal.expenses}_{kt}=\sum_{h} \bar{price}_{ht}\times{number of months in care}_{kht}$ | (S1) |
| --- | --- | --- |

where the price $\bar{price}_{ht}$ is the average monthly price for each type *h* of care, *t* is time (month and year) across all municipalities, and *number of months in care* is measured for municipality *k,* type *h* and at time t.

**Data imputation**

Comparing these “calculated expenses” with the actual expenses shows considerable differences, which reflects that there is some variation in prices of the same type of care across municipalities. For foster families, for example, the prices in 2016 vary between 4,681 Euro and 7,220 Euro across municipalities. The price difference can arise because of the composition of children across municipality and the organization of the foster families. To capture such variation between municipalities, we estimate a time-invariant factor for each municipality to adjust the level of expenses. We here exploited that we do have the actual expenses on an annual basis, which we compared with our measure based on average prices and number of months’ care. First, we construct an annual measure of calculated expenses relative to actual expenses:

|  | $F_{kt}=\frac{{cal. expenses}_{kt}}{{actual expences}_{kt}}$ | (S2) |
| --- | --- | --- |

Descriptive analyses (available upon request) show that there is a considerable variation in F between municipalities. Using this measure, we construct a municipality specific adjustment factor using the following two way fixed effect regression model

|  | $F_{kt}=\beta_{0}+\gamma_{t}+\mu_{k}+\epsilon_{kt}.$ | (S3) |
| --- | --- | --- |

Based on the regression model we find the predicted factor:

|  | $\hat{F}_{kt}=\hat{\beta}_{0}+\hat{\gamma_{t}}+\hat{\mu_{k}}$ | (S4) |
| --- | --- | --- |

We subsequently impute the monthly expenses at the municipal level as:

|  | ${Imp. Expenditure}_{kt}=\sum_{h} \frac{{price}_{kht}\times{months}_{kht}}{\hat{F_{kt}}}$ | (S5) |
| --- | --- | --- |

S2 Fig shows that there is a good fit between imputed and actual expenditures. The precision of the imputed expenditures can vary between years and between municipalities.

**S2 Fig. Plot of imputed expenditures and actual expenditures.**


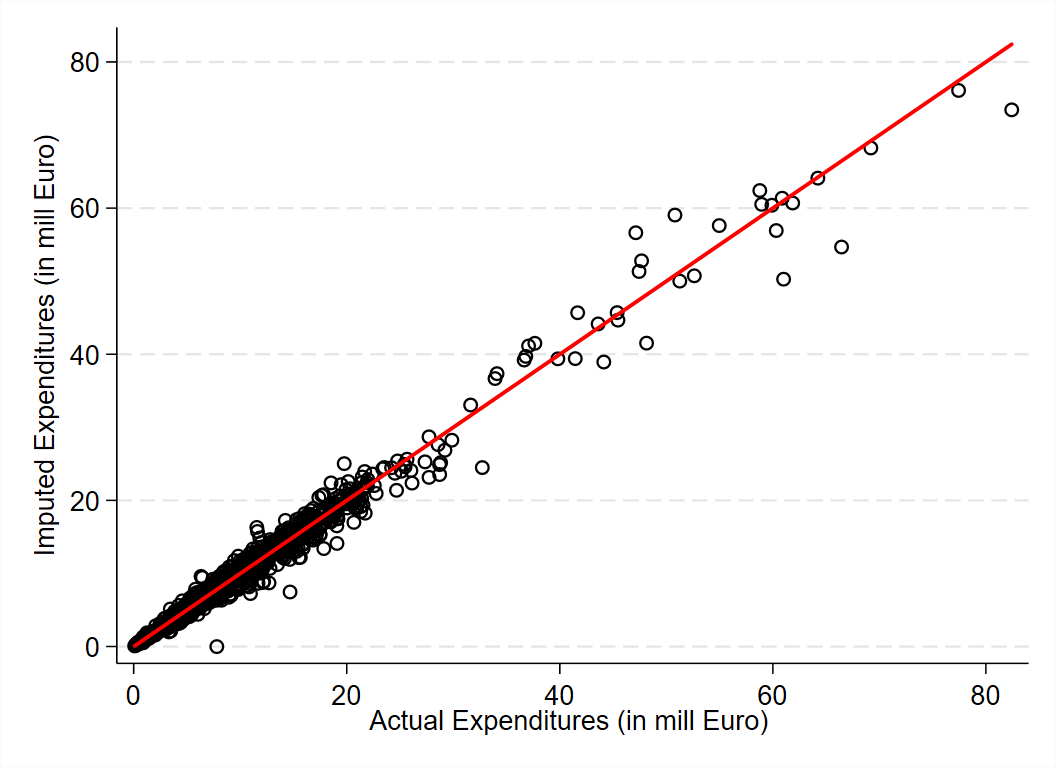


Each observation represents annual municipality expenditure. The solid red line represents the 45-degree line.

Based on imputed monthly expenses, we calculate the budget share each month *t*:

|  | $Budget share_{kt}=\frac{Imp.Expenses_{k,Jan}+..+Imp. Expenses_{kt}}{{Total annual budget}_{kt}}$. | (S6) |
| --- | --- | --- |

Our measure of monthly, imputed expenditures are calculated and based on average prices, which may give rise to measurement error in the budget share. Therefore, we expect an attenuation bias towards zero, and we will consider our estimate as a conservative estimate of the impact of the budget on child protection measures.

S3 Fig shows aggregate data for municipal budgets for out-of-home care. The mean municipal budget is around 12 million Euro. Naturally, budget sizes depend on municipality population size.

**S3 Fig. Municipality budgets for out-of-home care across percentiles.**


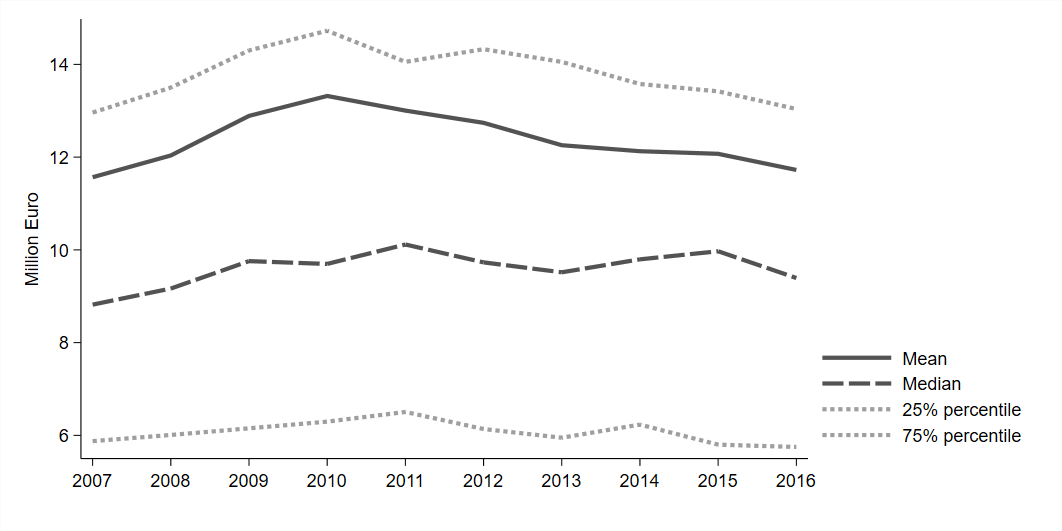


*Source:* The municipal budgets for out-of-home care. Annual observations for the period 2007-2016. Statistics Denmark, Statistikbanken, Table BUDK53.

S4 Fig shows average municipal budget overruns on out-of-home care before and after 2011 for the 98 Danish municipalities.

**S4 Fig. Budget overruns on out-of-home care in 2010 and 2011.**


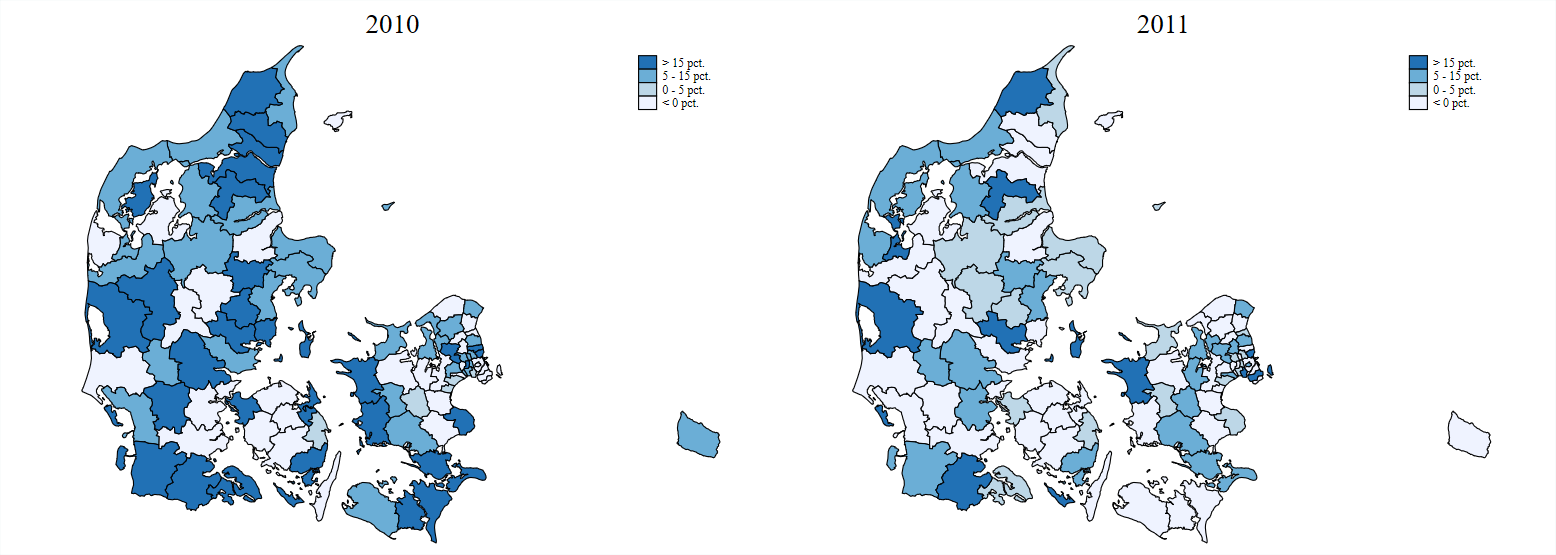


*Source:* Budget overrun is defined as the actual expenses on out-of-home care divided by the budget for out-of-home care. Own calculations based on data from Statistics Denmark, Statistikbanken, Table BUDK53 and REGK53.

S5 Fig shows average municipal budget overruns on eight selected budget items for the period 2007-2016. We created this data based on municipal budgets by comparing individual municipal budgets with ex-post municipal accounts in the same year. As noted, budget over- and underruns are prevalent in the period. The budget for out-of-home care suffered from budget overruns in the period 2007-2010, while budget overruns were reduced after 2010. The measure of budget overrun is particularly noisy for out-of-home care, indicating a large variance in out-of-home care budget deficits across municipalities over the entire period.

**S5 Fig. Average budget overruns for a selection of municipal activities.**


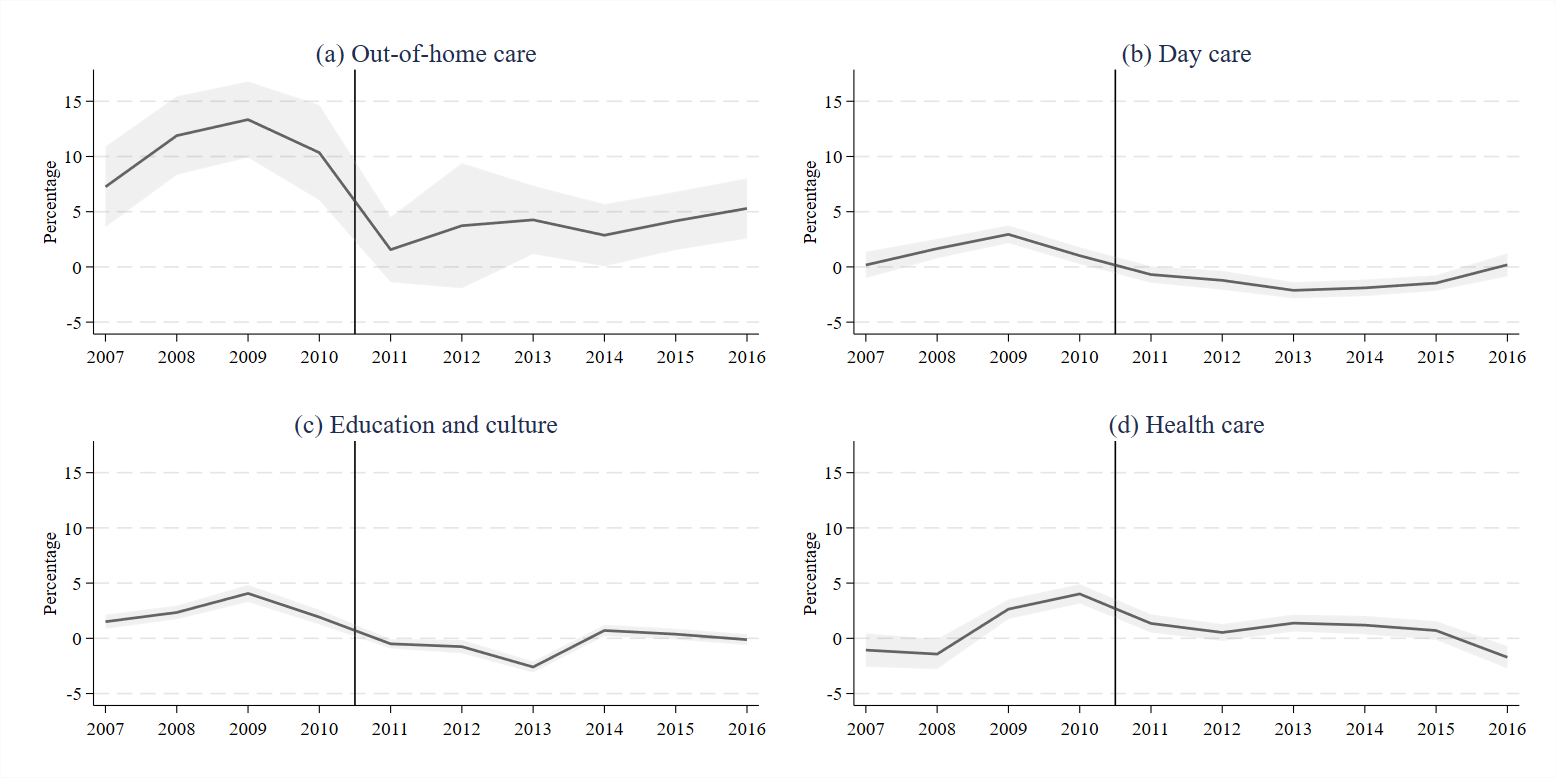


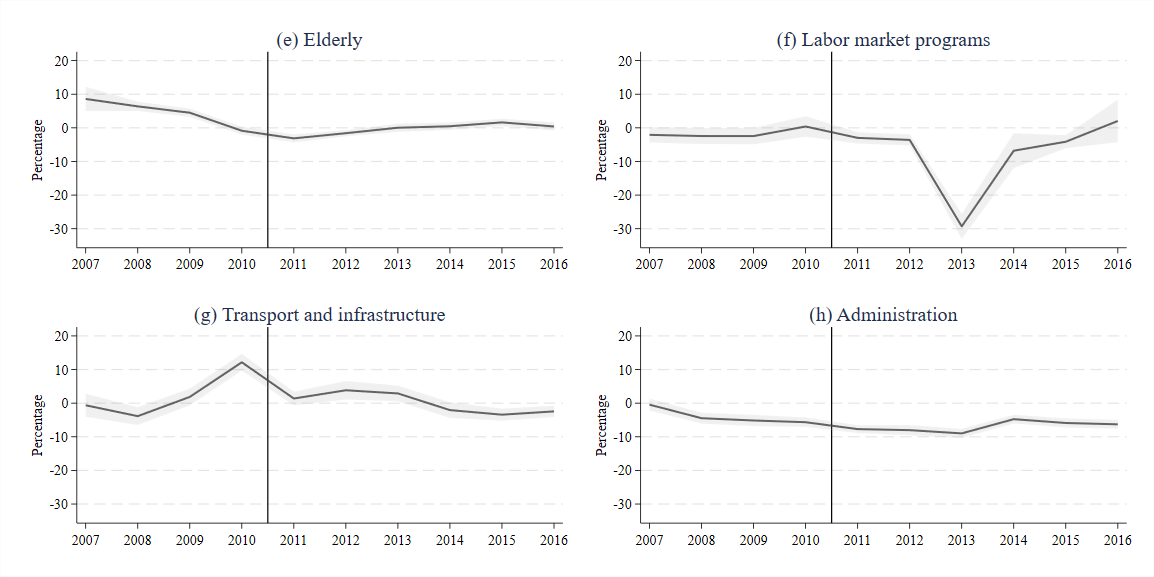


*Source:* The graphs shows the average of annual actual expenses divided by annual expenses in the budget for different items in the municipalities. Own calculations based on data from Statistics Denmark, Statistikbanken, Table BUDK53 and REGK53. The vertical line marks the introduction of sanctions for budget overruns.

**Outcome variables and control variables**

In our empirical analyses, we performed analyses at the municipal level and at the individual level. At the municipal level, the outcome variable is defined as the total number of children in care in each month.

When moving to the individual level, we examined how municipalities adjust their expenditures. Municipalities may adjust on five different margins when experiencing financial pressure on expenditures for at-risk children:

1. Municipalities may interrupt existing out-of-home care for children below age 18. If out-of-home care ends, children must either return to their biological parents (reunification) where other measures may initiated to help the family going forward. Reunification has direct and immediate budgetary consequences since it removes the expenditure for the out-of-home care.
2. Municipalities may end placement for children aged 18 or over. The municipality may choose to extend out-of-home care beyond age 18. However, at age 18 the child is legally an adult and the municipality’s legal responsibility for the child changes.
3. For children in preventive care programs, municipalities may choose to keep those children and their families in the preventive care program for a longer period rather than taking the step towards a much more expensive out-of-home care placement.
4. Municipalities may delay or completely give up new placements. This reaction will impede a further aggravation of the municipality’s financial distress. Municipalities may instead offer at-risk children a more inexpensive intervention, such as preventive care.
5. Municipalities can choose a cheaper type of out-of-home care when it initiates a placement, e.g. choose foster families instead of institutional care.

S2 Table depicts these five outcomes and the corresponding samples used to analyze the effects of budget shares for each outcome.

**S2 Table. Samples, outcomes, number of individuals and observations used in individual- level estimations, 2007-2016.**

| Sample | Selection | Outcome | # of individuals | # of observations |
| --- | --- | --- | --- | --- |
| 1 | Children below 18 in out-of-home care | Ending care  (re-unification) | 36,680 | 1,530,176 |
| 2 | Youth above 18 in out-of-home care | Ending care | 19,827 | 94,508 |
| 3 | Children below 18 in preventive action, but not in care | Out-of-home care the following month | 78,090 | 1,648,987 |
| 4 | Children below 18 ex ante neither in preventive nor out-of-home care, but will be the following month | Out-of home care (instead of preventive action) | 82,011 | 98,022 |
| 5 | Children below 18 where a new placement is initiated | Cheap (rather than expensive) placement* | 23,569 | 24,693 |
| *) Expensive placements are institutional care, secured institutional care, Social educational residency | | | | |

S3 Table reports the monthly means and standard deviations of the outcome variables and explanatory variables for these four samples. As evident from the table, the monthly “reunion rate” before the age of 18 is very low, whereas the reunion rate for child who have turned 18 is substantially higher, suggesting that three months after the 18th birthday half of the children are no longer in out-of-home care. We also find that the monthly transition rate from preventive actions to out-of-home care is low. This is because preventive care often functions as the first step to prevent an out-of-home placement. For some children, the situation improves following preventive care, and they will thus never go into out-of-home care, while other children are placed in out-of-home care if preventive action turns out not to be sufficient. For children who have not previously received preventive action, we see that more than 16 percent of the children will immediately go on to out-of-home care while around 84 percent of the children will start with preventive actions.

S3 Table shows summary statistics for each of the five individual-level samples. Notably, the samples differ in terms of average age, with children in out-of-home care being older. This is consistent with the fact that in the majority of cases preventive care measures are initiated before considering a more serious out-of-home care intervention. There are slightly more boys in all four samples. In general, parents of children in care or receiving preventive action have a weak labor market attachment, that is, a higher probability of being unemployed or outside the labor force. Compared to parents of children who have never been in out-of-home care, parents of children in care are less educated, more likely to be single, and have lower labor market income.

**S3 Table. Summary statistics, individual-level data.**

|  | (1) | (2) | (3) | (4) | (5) | (6) |
| --- | --- | --- | --- | --- | --- | --- |
|  | Re-union <18 | Re-union >18 | Placement from prevention | Placement vs. prevention | Cheap placement | No placement |
| Low birth weight | 0.0679 | 0.00575 | 0.0695 | 0.0555 | 0.0548 | 0.0353 |
|  | (0.252) | (0.0756) | (0.254) | (0.229) | (0.228) | (0.184) |
| Female | 0.455 | 0.424 | 0.416 | 0.449 | 0.466 | 0.489 |
|  | (0.498) | (0.494) | (0.493) | (0.497) | (0.499) | (0.500) |
| Age | 11.55 | 18.48 | 10.89 | 10.63 | 10.98 | 8.606 |
|  | (4.445) | (1.653) | (4.299) | (4.950) | (5.313) | (5.162) |
| Missing Mother | 0.0170 | 0.0283 | 0.00445 | 0.0125 | 0.0295 | 0.00267 |
|  | (0.129) | (0.166) | (0.0666) | (0.111) | (0.169) | (0.0516) |
| Missing Father | 0.0635 | 0.0670 | 0.0404 | 0.0527 | 0.0774 | 0.0182 |
|  | (0.244) | (0.250) | (0.197) | (0.223) | (0.267) | (0.134) |
| Immigrant Mother | 0.0894 | 0.107 | 0.152 | 0.157 | 0.127 | 0.119 |
|  | (0.285) | (0.309) | (0.359) | (0.364) | (0.333) | (0.323) |
| Descendant Mother | 0.00418 | 0.00175 | 0.00708 | 0.00772 | 0.00506 | 0.00844 |
|  | (0.0645) | (0.0417) | (0.0838) | (0.0875) | (0.0710) | (0.0915) |
| Mother’s labor inc. (mill DKK) | 0.270 | 0.331 | 0.271 | 0.229 | 0.320 | 0.531 |
|  | (0.444) | (0.470) | (0.445) | (0.420) | (0.467) | (0.499) |
| Mother outside labor force | 0.320 | 0.292 | 0.158 | 0.157 | 0.274 | 0.0384 |
|  | (0.467) | (0.455) | (0.365) | (0.364) | (0.446) | (0.192) |
| Mother unempl. | 0.141 | 0.0774 | 0.0729 | 0.0857 | 0.159 | 0.0198 |
|  | (0.348) | (0.267) | (0.260) | (0.280) | (0.366) | (0.139) |
| Mother single | 0.503 | 0.444 | 0.491 | 0.480 | 0.509 | 0.158 |
|  | (0.500) | (0.497) | (0.500) | (0.500) | (0.500) | (0.365) |
| Mother’s age | 31.79 | 35.65 | 36.70 | 36.16 | 31.61 | 31.32 |
|  | (16.28) | (19.11) | (12.33) | (12.25) | (16.39) | (16.45) |
| **Mother’s education:** |  |  |  |  |  |  |
| Primary school | 0.593 | 0.468 | 0.403 | 0.407 | 0.499 | 0.138 |
|  | (0.491) | (0.499) | (0.490) | (0.491) | (0.500) | (0.345) |
| Secondary educ. | 0.220 | 0.269 | 0.357 | 0.343 | 0.287 | 0.343 |
|  | (0.414) | (0.443) | (0.479) | (0.475) | (0.452) | (0.475) |
| **Outcome variables:** |  |  |  |  |  |  |
| Reunion | 0.00571 | 0.206 |  |  |  |  |
|  | (0.0754) | (0.405) |  |  |  |  |
| Placement |  |  | 0.00447 | 0.158 |  |  |
|  |  |  | (0.0667) | (0.364) |  |  |
| Cheap type |  |  |  |  | 0.519 |  |
|  |  |  |  |  | (0.500) |  |
| Observations | 1,530,176 | 94,508 | 1,648,987 | 98,022 | 24,693 | 12,840,842 |
| N | 36,680 | 19,827 | 78,090 | 82,011 | 23,569 | 1,989,925 |

On samples in each column, see S2 Table. Column (1) represents Sample 1, column (2) Sample 2, column (3) Sample 3, column (4) Sample 4, column (5) Sample 5, and column (6) the entire population of 0-17 year-old children.

**Additional results**

The following section presents additional estimation results, which complement the results presented in the paper. Moreover, we present the results from a number of robustness checks of the main analysis. S6 Fig shows the effects of budget overrun for the five outcomes, before and after 2011. The effects of budget overrun are stronger and more significant for most outcomes.

**S6 Fig. Marginal effects of budget share on child protection measures, before/after 2011.**


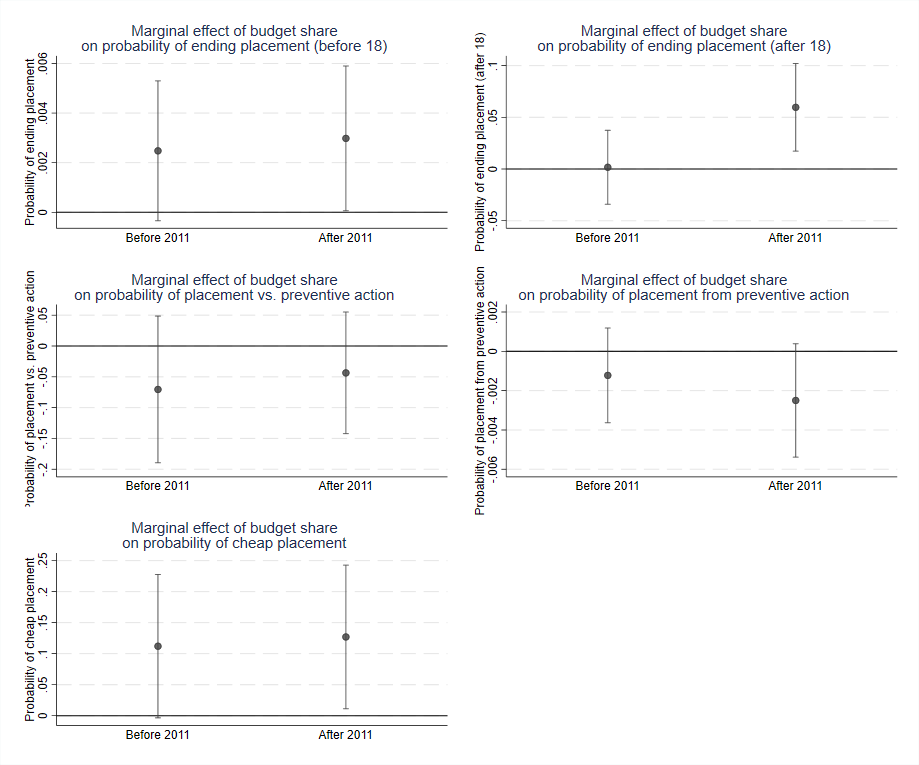


Top Panel: Predicted probability of ending a placement for a youth above 18 in the month of July as a function of the budget share at July 1^st^. Middle Panel: Predicted probability of a placement instead of preventive action for a child below 18 in the month of July as a function of the budget share at July 1^st^. Bottom Panel: Predicted probability of initiating a cheap placement (instead of an expensive placement) for a child below 18 in the month of July as a function of the budget share at July 1^st^. Confidence intervals at 0.05 level. Standard errors clustered at municipal level.

**S4 Table. Estimation results, individual micro data based regression.**

|  | (1) | (2) | (3) | (4) | (5) |
| --- | --- | --- | --- | --- | --- |
|  | Re-union <18 | Re-union >18 | Placement from prevention | Placement vs. prevention | Cheap placement |
|  | b/se | b/se | b/se | b/se | b/se |
| lag budget | 0.4369 | 0.0156 | -0.2624 | -0.3695 | 0.5377 |
| before 2011 | (0.2578) | (0.1685) | (0.2631) | (0.3189) | (0.2832) |
| lag budget | 0.5227^*^ | 0.4487^**^ | -0.4977 | -0.2780 | 0.6078^*^ |
| after 2011 | (0.2571) | (0.1627) | (0.2948) | (0.3220) | (0.2822) |
| Girl | 0.0270 | 0.0926^***^ | 0.1184^***^ | 0.2015^***^ | 0.2802^***^ |
|  | (0.0269) | (0.0169) | (0.0313) | (0.0214) | (0.0265) |
| Age | -0.2247^***^ | -27.1900^***^ | -0.1435^***^ | -0.2597^***^ | -0.2690^***^ |
|  | (0.0142) | (0.4902) | (0.0156) | (0.0107) | (0.0149) |
| Age squared | 0.0156^***^ | 0.6894^***^ | 0.0100^***^ | 0.0136^***^ | 0.0103^***^ |
|  | (0.0007) | (0.0126) | (0.0007) | (0.0006) | (0.0009) |
| Missing info, mother | 0.2890^*^ | 0.0626 | 0.1034 | 0.9431^***^ | -0.5782^***^ |
|  | (0.1305) | (0.0729) | (0.2454) | (0.1484) | (0.1425) |
| Missing info, father | -0.1850^**^ | 0.0114 | 0.0497 | 0.4126^***^ | 0.1880^**^ |
|  | (0.0573) | (0.0585) | (0.0675) | (0.0460) | (0.0650) |
| **Information mother** | |  |  |  |  |
| Immigrant | 0.5291^***^ | -0.1528^***^ | -0.2519^**^ | -0.0933^*^ | -0.3998^***^ |
|  | (0.0542) | (0.0406) | (0.0768) | (0.0389) | (0.0845) |
| Descendant | 0.3052 | 0.3144^***^ | -0.7768^**^ | -0.0736 | -0.4273^*^ |
|  | (0.2282) | (0.0891) | (0.2684) | (0.1530) | (0.2175) |
| Labor income | 0.1137^***^ | 0.0867^**^ | -0.2447^***^ | -0.0852^**^ | -0.3074^***^ |
|  | (0.0312) | (0.0325) | (0.0602) | (0.0312) | (0.0487) |
| Outside labor | -0.2612^***^ | 0.0566 | -0.0023 | 0.0410 | 0.1500^*^ |
| force | (0.0558) | (0.0396) | (0.0628) | (0.0463) | (0.0670) |
| Social security | 0.3152^***^ | -0.0622 | 0.2711^***^ | 0.1388^**^ | -0.0961 |
|  | (0.0484) | (0.0458) | (0.0509) | (0.0454) | (0.0634) |
| Single parent | 0.0896^**^ | -0.0014 | 0.2128^***^ | -0.0307 | 0.2765^***^ |
|  | (0.0278) | (0.0206) | (0.0348) | (0.0236) | (0.0270) |
| Age, mother | 0.0207^***^ | -0.0040 | -0.0227^***^ | -0.0224^***^ | -0.0306^***^ |
|  | (0.0047) | (0.0031) | (0.0060) | (0.0047) | (0.0054) |
| Age, mother, | -0.0002^***^ | 0.0001 | 0.0001 | 0.0001 | 0.0002^*^ |
| squared | (0.0001) | (0.0001) | (0.0001) | (0.0001) | (0.0001) |
| Primary | -0.1826^***^ | -0.0083 | 0.2764^***^ | 0.2985^***^ | 0.3818^***^ |
| school | (0.0327) | (0.0326) | (0.0495) | (0.0339) | (0.0489) |
| Secondary | 0.0411 | 0.0315 | 0.0849 | -0.0152 | 0.1757^***^ |
| school | (0.0353) | (0.0306) | (0.0444) | (0.0342) | (0.0480) |
| Low birth | -0.2868^***^ | 0.0533 | -0.0845 | -0.0472 | -0.1381 |
| weight | (0.0592) | (0.0634) | (0.0552) | (0.0392) | (0.0882) |
| Constant | -5.6557^***^ | 262.3477^***^ | -6.2091^***^ | -0.2779^*^ | -0.8061^***^ |
|  | (0.0926) | (4.7001) | (0.1773) | (0.1326) | (0.1419) |
| Month dummies | Yes | Yes | Yes | Yes | Yes |
| Year dummies | Yes | Yes | Yes | Yes | Yes |
| Municipal dummies | Yes | Yes | Yes | Yes | Yes |
| N | 1,530,086 | 94,637 | 1,200,258 | 70,340 | 24,305 |
| R^2^ |  |  |  |  |  |

On samples in each column, see S2 Table. Column (1) represents Sample 1, column (2) Sample 2, column (3) Sample 3, column (4) Sample 4, column (5) Sample 5.

**Heterogeneous effects**

As a supplement to the analyses presented in the paper, we tested whether some municipalities were more likely to react to budget concerns than others. We investigated 12 selected municipality characteristics that we hypothesize may affect municipal decisions regarding at-risk children. The characteristics relate either to the composition of the citizens of the municipality or the political and economic environment of the municipality. The five compositional characteristic include population size, fraction of citizens with only primary education, fraction of single parents, average income per capita, the number of children in care by January 1^st^ 2007. The four political characteristics include whether the whether the majority in the municipality council consisted of parties on the center-left, whether the city council consisted of more than 30 percent women, the year is an local election year and if the majority in the municipality council belongs to the same political coalition as the prime minister. Finally, we have three characteristics that relate to the financial circumstances of the municipality: if the municipality overrun the budget the previous year, the average municipal debt per capita and an index for the financial resources (resource pressure) needed for citizens in the municipality. All the characteristics are made into dummy variables. For the compositional characteristics the dummy variable is based on the median e.g. if the average income per capita is above median average income per capita. As shown in S5 Table, none of these municipality characteristics matter for the size of the effect.

**S5 Table. Heterogeneity test, individual micro data based regression.**

|  | Re-union before 18 | Re-union after 18 | New placement | Placement from prevent. action | Cheap placement |
| --- | --- | --- | --- | --- | --- |
| Budget overrun |  |  |  |  |  |
| Test statistic | 4.054 | 1.980 | 0.272 | 0.296 | 0.432 |
| P-value | 0.132 | 0.371 | 0.873 | 0.862 | 0.806 |
| Red block |  |  |  |  |  |
| Test statistic | 5.859 | 2.467 | 0.452 | 0.092 | 0.634 |
| P-value | 0.053 | 0.291 | 0.798 | 0.955 | 0.728 |
| Population size |  |  |  |  |  |
| Test statistic | 0.627 | 1.194 | 0.492 | 1.297 | 4.550 |
| P-value | 0.731 | 0.550 | 0.782 | 0.523 | 0.103 |
| Women in municipal councils |  |  |  |  |  |
| Test statistic | 0.863 | 0.433 | 3.605 | 7.391 | 3.231 |
| P-value | 0.650 | 0.805 | 0.165 | 0.025 | 0.199 |
| Highest education primary school |  |  |  |  |  |
| Test statistic | 0.988 | 0.011 | 0.119 | 3.783 | 4.266 |
| P-value | 0.610 | 0.995 | 0.942 | 0.151 | 0.118 |
| Share of single parent |  |  |  |  |  |
| Test statistic | 1.965 | 1.005 | 1.575 | 1.436 | 0.318 |
| P-value | 0.374 | 0.605 | 0.455 | 0.488 | 0.853 |
| Average Income |  |  |  |  |  |
| Test statistic | 0.254 | 2.444 | 2.525 | 0.532 | 5.246 |
| P-value | 0.881 | 0.295 | 0.283 | 0.766 | 0.073 |
| 100+ placements Jan. 1, 2007 |  |  |  |  |  |
| Test statistic | 3.066 | 0.068 | 2.775 | 0.918 | 2.254 |
| P-value | 0.216 | 0.966 | 0.250 | 0.632 | 0.324 |
| Debt per capita > 25000 DKK |  |  |  |  |  |
| Test statistic | 3.302 | 18.598 | 1.754 | 0.535 | 0.641 |
| P-value | 0.192 | 0.000 | 0.416 | 0.765 | 0.726 |
| Election year |  |  |  |  |  |
| Test statistic | 2.094 | 4.334 | 0.478 | 4.125 | 6.635 |
| P-value | 0.351 | 0.115 | 0.787 | 0.127 | 0.036 |
| Ressource press |  |  |  |  |  |
| Test statistic | 0.746 | 1.670 | 3.541 | 0.583 | 3.293 |
| P-value | 0.689 | 0.434 | 0.170 | 0.747 | 0.193 |
| Majority of municipal council same block as prime minister |  |  |  |  |  |
| Test statistic | 8.712 | 0.196 | 1.129 | 0.329 | 0.305 |
| P-value | 0.013 | 0.906 | 0.569 | 0.848 | 0.858 |

The table shows the results of the logit estimation relating to equation (3), with an interaction term between the municipality characteristic a dummy for after 2011 and the budget share. The test statistics and the p-value refer to the F-test statistics for the null hypothesis that there is no heterogeneity in terms of the municipality characteristic. For a description of the municipality characteristics see the text above the table.

**S6 Table. Heterogeneity test, municipality-level analysis.**

|  | Baseline |
| --- | --- |
| Budget overrun |  |
| P-value | 0.335 |
| Red block |  |
| P-value | 0.257 |
| Population size |  |
| P-value | 0.196 |
| Women in municipal councils |  |
| P-value | 0.989 |
| Highest education primary school |  |
| P-value | 0.317 |
| Share of single parent |  |
| P-value | 0.368 |
| Average Income |  |
| P-value | 0.925 |
| 100+ placements January 1st 2007 |  |
| P-value | 0.093 |
| Debt per capita > 25000 DKK |  |
| P-value | 0.260 |
| Election year |  |
| P-value | 0.262 |
| Ressource press |  |
| P-value | 0.480 |
| Majority of municipal councils same block as prime minister |  |
| P-value | 0.526 |

The table refers to the estimation of equation (2) including an interaction term between the municipality characteristic interacted with lag budget share. The municipality characteristic described in the text above the table. The p-value refers to the test for the null hypothesis of no heterogeneity in terms of the municipality characteristics.

**Placebo tests**

Finally, we performed a robustness test to check whether our results could be driven by the way municipalities organize their work with at-risk children over the (budget) year. We thus ran placebo tests, where we constructed “placebo” data, pretending that the fiscal year runs from July to June (instead of from January through December, as is the actual situation). We constructed “placebo” monthly budgets by defining a fiscal “annual” budget as the average of the budget for the two calendar years. We next constructed measures of monthly expenditures as before and then constructed a placebo cumulated budget share for each month in the placebo fiscal year starting July 1 and ending June 30 the following year (see S7 Fig).

**S7 Fig. Placebo budget share.**


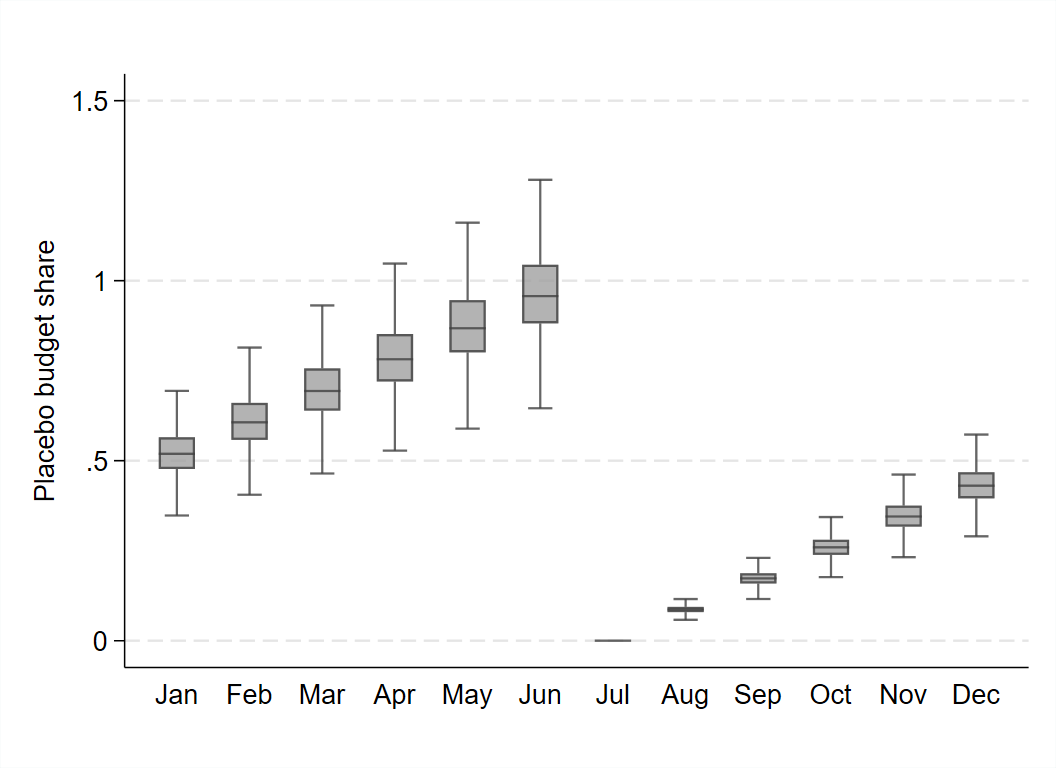


The placebo budget share is constructed for a “placebo” fiscal year that we pretend runs from July one year to June the following year. The placebo budget share is measured at the beginning of the month. For July, the placebo budget share is always zero. For August, the placebo budget share is defined as the proportion of the total placebo budget (the average of the two years around) for year *t* that was used in July. For September, the placebo budget share is calculated as the share of July and August expenditure out of the total budget, etc. Finally, for June the following year, placebo budget share is defined as expenditure share of months July-May in the two years.

We then ran exactly the same estimations as in our main specifications with the placebo budget share instead of the actual budget share. The result of the placebo test for the analysis on municipality level is shown in Table 1 (main paper), last column. The results show that effect of the placebo budget is share is very small and positive, which indicates that the effect of the true budget share is not a result of mean reversion.

For the individual-level specifications, we ran the placebo test for the two individual outcomes that in the main analysis showed statistically significant effects. The results of these placebo tests are shown in S8 Fig. They show that the effects of the budget share on the probability of ending out-of-home care for children below and above age 18, and on the probability of choosing a cheaper placement are now insignificant and the coefficients are numerically smaller. Thus, the individual-level placebo tests also suggest that mean reversion is not driving out main results.

**S8 Fig. Estimation results from placebo tests.**

**Placebo test Actual results**

**
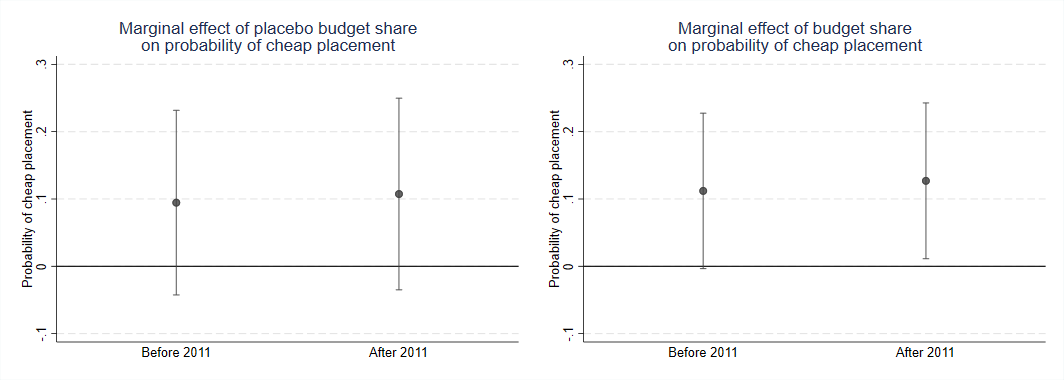
**

**
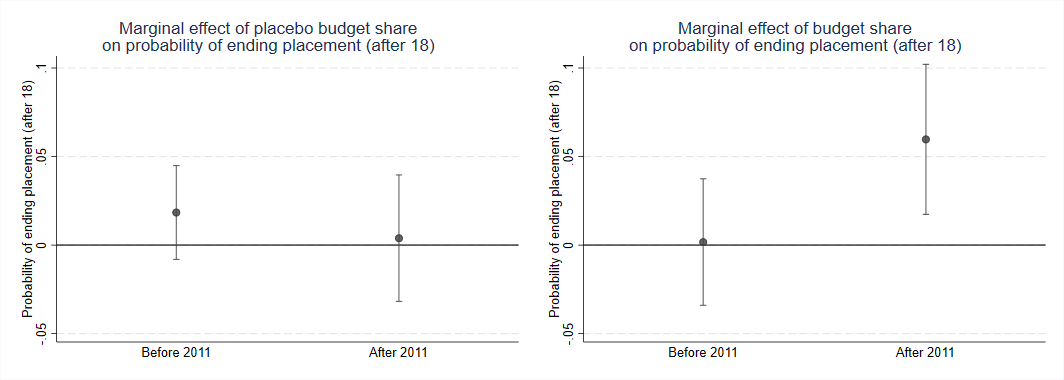
**

**
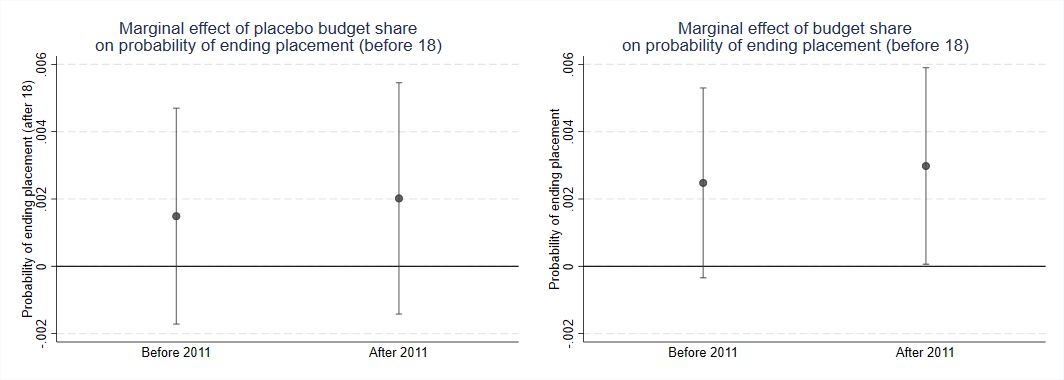
**

The first row shows analysis of ending the placement after turning 18 and the second row the analyses of choosing a foster care instead of preventive action. The left column shows the placebo test and the right column the actual analyses.
