# Supplementary material for: Trading off fiscal budget adherence and child protection
Source: PLoS One. 2022 Mar 24;17(3):e0261664. doi: 10.1371/journal.pone.0261664 (PMC8947134; doi:10.1371/journal.pone.0261664)
Supplement: Attachment — Submitted filename: report_PONE_D_21_06271.pdf [file pone.0261664.s002.pdf]

Trading off fiscal budget adherence and child protection

Referee Report

April 11, 2021

This paper addresses the link between fiscal constraints and investments/management of child protection programs, using danish data. The underlying idea is that, if you impose fiscal constraints to lower levels of government, you might end up having side effects, as the ones detected on the allocation of resources for child protection. Having granular data. the authors can disentangle the use of money in the first part of the year, and they exploit the differences between those municipalities having spent the majority of their budget in child protection by July 1st (high spending) and those not having spent the majority of their budget in child protection by July 1st (low spending), to assess the role of budget constraints introduced starting 2011 in Denmark.

While the topic is extremely interesting, I find several conclusions reached by the authors as unsubstantiated and therefore needy of more support. I provide some suggestions in this regard, not in the order of importance.

1. I found the takeaway of the paper a little bit naive in terms of policy implications. It basically proposes that there should be no budget constraints and municipality should be set free to overrun any expenditure limits. Yet, even if this should be the case, resources are scarce, even waiving the EU stability pact. This means that choices would be needed on which item to allocate the scarce financial resources available in the municipal budget. At that point we would need a criterion to prioritize resources allocation, which does not emerge by the authors' analysis. Another scenario could be that municipalities could raise their own taxes to increase revenues so that they could fuel child protection vis a vis other welfare programs. However, this scenario should address the possibility to "vote with the feet", which stands for within a country migration. Hence, I do think that the paper would substantially benefit from a clear theoretical framework at the beginning spelling out which kind of institutional setting the authors have in mind.

2. Related to the previous point and the policy implications: the claim at the end of the paper "potentially harmful consequences" is not proven. The authors show that there is a decrease in certain actions related to child-protection programs, but they do not bring any evidence that this reduction is associated to an increase in social distress for the part of the population most interested by the program. Are high school drop-outs

in those municipalities increasing? Is the rate of police interventions for child neglecting increasing? Are there more emergency room recoveries for the population below 18? I do think this is pivotal to assess the relevance at the society level of these cuts or reduction in the use of child protection measures.

3. A final remark on policy implications: the difference between day care and child protection is not just a matter of the structure of the costs (as suggested at p.26) but it is also a matter of the proportion of residents affected by the service. Using the descriptive stats, the authors should provide a flavor of how relevant are the population groups more likely to be interested by child protection in a given municipality in a given year. For instance, how relevant are single mums in a certain municipality, or immigrants? They could use these infos to run specific heterogeneities.

4. The measure of taking kids out of their home is an extreme ratio. There might be early intervention programs in order to avoid this extreme measure. Has anything happen in that respect during the observational period? It appears that there has been a decrease in the use of out-of-home procedure independent from the 2011 reform (see Figure A5), could it be due to the use of alternative solutions?

5. As for the empirical approach, why do not adopt a more simple scenario. Split municipalities–using an aggregated measure referred to the period before the reform–into more and less fiscal conservative (so to say, more or less available to run over expenditures). For instance using the average excess budget run in the pre-reform period. Then you know that the introduction of the 2011 reform will have a different intensity, and you can check the effect of the intensity of the treatment on child-protection actions and expenditures. I find the use of the expenditures up to July 1st as an extra, rather than an intuitive baseline.

6. As an extra heterogeneities I would consider the alignment between the local and the national government (i.e. more funding or more flexibility), and to consider the distance from the election month, which could affect spending decisions (conditional to the relevance of the affected population).
