# Supplementary material for: Trading off fiscal budget adherence and child protection
Source: PLoS One. 2022 Mar 24;17(3):e0261664. doi: 10.1371/journal.pone.0261664 (PMC8947134; doi:10.1371/journal.pone.0261664)
Supplement: Attachment — Submitted filename: 20200414_PLOSONE_budgetchildprotection.pdf [file pone.0261664.s003.pdf]

Referee Report on "Trading off fiscal budget adherence and child protection" for PLOS ONE

Summary: This paper studies the effect of local government budget constraints on child protection decisions. Using data from Denmark, they find that areas that are over-budget for the first-half of the year reduce the number of children in care in the second half of the year, primarily by ending placements for individuals who are 18 or older. They are also more likely to opt for less expensive placements.

Data availability note: The data cannot be made available as it is Danish register data. The authors say they will make all code available.

Major comments:

1. I do not have major comments as the paper seems technically sound. I do think it would potentially benefit from looking at other thresholds other than 60% of budget used halfway through the year. This could either be as robustness or to show a change in the effect by the amount of overrun. At a minimum, there needs to be some discussion about why the 60% threshold was used instead of something else.

Minor comments:

1. Figures and tables need some additional notes in order to be self-contained. In particular, Figure 1, Table 1, and Figure 4 all need more explanation so that a reader could flip to them and understand them without referencing the text.
2. The models in Table 1 need column numbers (1), (2), (3).
3. Authors refer multiple times to using a lag of the dependent variable in the regression. However, the dependent variable is change in log children, while the independent variable is log children, so it is not the lag of the dependent variable. This language needs to be adjusted to accurately reflect what is in the model. I am not as familiar with the term ADL model, but I think this term may not be accurate for the same reason.
4. I think it is worth mentioning that these results likely reflect a lower bound on the effect of budget constraints on placements since budget constraints are likely to affect decisions earlier in the year as well. They just become more salient and more of the uncertainty has been realized later in the year.
5. I think Figure 2 would be more useful if it was shown as a ratio of the actual budget spent to the expected budget spent. An alternative may also be to add a dotted line for expected fraction of budget spent (assuming the same fraction is spent in each month). Either would make it easier to tell how far off the numbers are from their expected values.
6. On page 12, when explaining the equation (2), it would be useful to explain who is included in each regression. So, the first one would include all children in care, presumably. The second one must include children at-risk for care by some measure, and I am not sure about the third.
7. I was surprised the interaction with post-2011 was not significant. Do the authors have any theories about that?
8. I thought the placebo test was good and useful.
9. On page 20, the authors say "The problem may be particularly relevant for small municipalities with fewer options for cost smoothing." This is a testable hypothesis, so I would suggest that the authors test it in the data and report the findings.

Very Minor comments:

1. Compound adjectives need hyphens throughout the paper. For example: individual-level, low-spending, long-run, etc. all need hyphens when they are used as adjectives. Two-thirds and one-third also need hyphens.
2. Page 4, last sentence of the first paragraph: delete "which" and delete the apostrophe after citizens.
3. Early in the paper, it is mentioned which placements are more expensive and which less expensive. It would be helpful to provide a footnote later with this information for quick reference when it is mentioned again in the results (page 16, for example).
4. Page 17, line 353 should be "0.5 percentage point"

Thanks! It was an interesting read.
