# Supplementary material for: Trading off fiscal budget adherence and child protection
Source: PLoS One. 2022 Mar 24;17(3):e0261664. doi: 10.1371/journal.pone.0261664 (PMC8947134; doi:10.1371/journal.pone.0261664)
Supplement: Attachment — Submitted filename: report_PONE_D_21_06271_revision.pdf [file pone.0261664.s005.pdf]

# Trading off fiscal budget adherence and child protection

Referee Report

November 14, 2021

I appreciate the attempts of the authors to address all the raise concerns. Yet I do think they have to turn down the selling point on "We thus highlight that enforcing strict budget adherence can have unintended and potentially devastating side effects." They do show that there is a trade off and that there is a decrease in the outcome of interest, but they do not have a smoking gun on the cognitive performance or emotional response in the treated children. I would suggest only to be open to that in their narrative.
