# Supplementary material for: Trading off fiscal budget adherence and child protection
Source: PLoS One. 2022 Mar 24;17(3):e0261664. doi: 10.1371/journal.pone.0261664 (PMC8947134; doi:10.1371/journal.pone.0261664)
Supplement: Attachment — Submitted filename: Response to reviewers_August_13.docx [file pone.0261664.s004.docx]

**Response to the editor and reviewers**

**Response to the editor**

Thank you for providing a number of constructive and insightful comments and suggestions. We have tried our best to respond to these in the response letter and to incorporate the suggestions in the paper to the extent possible. We think that the comments and suggestions received have significantly improved the revised version of the paper.

Below we address the comments and suggestions one by one. Our responses are marked by using indented blue font.

1. The first major concern is related to the lack of any evidence that reducing spending leads to adverse effects on children.
   a. Such evidence and discussion should be included in the revised version

*Response: Thank you for raising this issue. We completely agree that it would strengthen our case if we could provide evidence on the potential harmful effects on children if child protection is influenced by budget concerns. We have been looking for outcomes for children where we could investigate the potential harmful consequences. Our challenge is that age of the children in our sample varies from 0-17 and it is difficult to find relevant outcomes. In our data, we have educational outcomes like completion of primary school, high school and vocational education. We have performed an analysis for a subsample of the children who are in out-of-home placement just before their 18th birthday as our results indicate that risk of budget overrun increases the likelihood of ending an out-of-home placement at 18. We investigate if the budget share affects the likelihood of completion of high school/vocational training by the age of 21. We do find evidence that after 2011 the probability of completion is lower for children in care in municipalities in risk of budget overrun, but the effect is not significantly different from zero. We enclose the results below in Table B1. We have chosen not to include these new results in paper, as the results are not significant. Although we are not with our current data able to credibly show harmful effects on child outcomes of budgetary concerns, we still think it is important to document that budget adherence may have unintended side-effects in the sense that child protection decisions are influenced by factors such as (within-year variation in) budget adherence, which is against the intention behind child protection laws and principles.*

b. as well as clarifying the theoretical framework as suggested by Reviewer 1.

*Response: We have chosen to emphasize the discussion of fiscal federalism theory as our framework for the empirical model and estimations. Specifically, we have rearranged the introduction by moving around sections and adding more discussion that clarifies our approach. We thus start the introduction by discussing the challenges of curbing public expenditure and the role of local government budgets. After that, we go on to discuss the main insights from the literature on fiscal federalism. Finally, we discuss municipal activities towards at-risk children as a relevant case to illustrate how variation in budget pressure over the months in the fiscal year may impact decisions and prioritizations regarding public services.*

*In the same way, we have moved around paragraphs in section 2 to emphasize the institutional setup related to budgetary delegation. Finally, we have edited the Discussion and conclusion section (primarily the first and the last paragraphs) to be more precise about our focus on the variation in budget overrun over months within a fiscal year.*

2. The second major concern has to do with the empirical strategy which is not always clearly presented and the discussion of the findings is sometimes incomplete.
   a. Reviewer 1 suggests an alternative way of assigning municipalities into low or high risk of exceeding their budget based on their budget history before the reform of 2011, which is worth exploring.

*Response: We have tried to sharpen our presentation of the empirical strategy. Specifically, we have tried to follow Reviewer 1’s suggestion and calculated a measure of intensity using the pre-reform data. We use data on annual budget overrun defined as actual expenses divided by expenses in the budget for years 2007-2010 to construct the intensity of the reform. For each municipality, we calculate average budget overrun for the pre-reform. We then use the post reform data (2011-2016) and investigate the impact of the intensity on the number of children in care. We do find strong and negative effects of the intensity on the number of children in care (see the Table B2 below). Notice that in this analysis we only have variation in the intensity across municipalities and can therefore not control for municipality fixed effects. This is the main reason for why we have chosen not to show these results in the paper, as we suspect unobserved municipality factors such as the number of children at risk in the municipality that may be correlated with the intensity measure.* *Our current approach allows us to exploit both within and across municipality variation, whereas the suggested approach would leave us only with across municipality variation. We see a lot of variation in budget shares within municipalities across years and it is not clear that some municipalities are always high- or low-spending. For these reasons, we do not think that the suggested approach would yield any additional information. We have adjusted the text to underline that we exploit both within and across municipality variation.*

b. Reviewer 3 is concerned with the individual-level analysis pointing correctly that there is no variation across individuals within a municipality. Indeed, equation (2) seems to suggest that the budget share of municipality (k) varies at the level of individual (i), which is not the case. As a result the analysis could easily be focused at the municipality level unless a convincing argument can be made for the individual-level analysis.

*Response: We agree that in principle, the analysis could be performed at the municipality level. However, in that case, we would have to weigh each municipality-level variable using a weight based on number of children in care or other weight. Moreover, we observe (Table A4) that individual-specific factors are important in decisions to place in out-of-home care. Thus, controlling for these individual factors strengthens our identification of municipal-level variation in budgets. Furthermore, we control for municipality-specific effects and cluster standard-errors at the municipal level.*

c. Reviewer 2 suggest to motivate and consider different cutoffs than the 60% used.

*Response: The 60% threshold is used in the presentation of descriptive evidence in Figure 3. We have now checked that the overall picture conveyed in Figure 3 is indeed robust to the specific choice of threshold (see Figure B1, below).*

*Moreover, we would like to add that the main analysis is not based on any particular threshold, but we gather from your comments that this is not all that clear in the text. We have adjusted the text, in particular the section describing the empirical strategy, to better reflect the fact that our results do not depend on any particular threshold. Thank you for making us aware of this.*

**Response to Reviewer #1**

Thank you for providing a number of constructive and insightful comments and suggestions. We have tried our best to respond to these in the response letter and to incorporate the suggestions in the paper to the extent possible. We think that the comments and suggestions received have significantly improved the revised version of the paper.

Below we address the comments and suggestions one by one. Our responses are marked by using indented blue font.

1. I found the takeaway of the paper a little bit naive in terms of policy implications. It basically proposes that there should be no budget constraints and municipality should be set free to overrun any expenditure limits. Yet, even if this should be the case, resources are scarce, even waiving the EU stability pact. This means that choices would be needed on which item to allocate the scarce financial resources available in the municipal budget. At that point we would need a criterion to prioritize resources allocation, which does not emerge by the authors' analysis. Another scenario could be that municipalities could raise their own taxes to increase revenues so that they could fuel child protection vis a vis other welfare programs. However, this scenario should address the possibility to "vote with the feet", which stands for within a country migration. Hence, I do think that the paper would substantially benefit from a clear theoretical framework at the beginning spelling out which kind of institutional setting the authors have in mind.

*Response: Thank you for this comment. Our intention was not to argue that fiscal restraint in general is problematic, but just to discuss the problems of delegating to the lowest administrative level when it comes to a task that is both very costly, is not in high demand by citizens in municipalities (because services are provided to a small and weak group where a Tiebout type “voting with their feet” will be largely unnoticed – or even cheered by municipality), and where the possible positive effects of intervention will only be seen in the very long run where the children may be citizens and hence taxpayers in another municipality.*

*In the revised version, we have chosen to emphasize the discussion of fiscal federalism theory as our framework for the empirical model and estimations. Specifically, we have rearranged the introduction by moving around sections and adding more discussion that clarifies our approach. We thus start the introduction by discussing the challenges of curbing public expenditure and the role of local government budgets. After that, we go on to discuss the main insights from the literature on fiscal federalism. Finally, we discuss municipal activities towards at-risk children as a relevant case to illustrate how variation in budget pressure over the months in the fiscal year may impact decisions and prioritizations regarding public services.*

*In the same way, we have moved around paragraphs in section 2 to emphasize the institutional setup related to budgetary delegation. Finally, we have edited the Discussion and conclusion section (primarily the first and the last paragraphs) to be more precise about our focus on the variation in budget overrun over months within a fiscal year.*

2. Related to the previous point and the policy implications: the claim at the end of the paper “potentially harmful consequences" is not proven. The authors show that there is a decrease in certain actions related to child-protection programs, but they do not bring any evidence that this reduction is associated to an increase in social distress for the part of the population most interested by the program. Are high school drop-outs in those municipalities increasing? Is the rate of police interventions for child neglecting increasing? Are there more emergency room recoveries for the population below 18? I do think this is pivotal to assess the relevance at the society level of these cuts or reduction in the use of child protection measures.

*Response: Thank you for raising this issue. We completely agree that it would strengthen our case if we could provide evidence on the potential harmful effects on children if child protection is influenced by budget concerns. We have been looking for outcomes for children where we could investigate the potential harmful consequences. Our challenge is that age of the children in our sample varies from 0-17 and it is difficult to find relevant outcomes. In our data, we have educational outcomes like completion of primary school, high school and vocational education. We have performed an analysis for a subsample of the children who are in out-of-home placement just before their 18th birthday as our results indicate that risk of budget overrun increases the likelihood of ending an out-of-home placement at 18. We investigate if the budget share affects the likelihood of completion of high school/vocational training by the age of 21. We do find evidence that after 2011 the probability of completion is lower for children in care in municipalities in risk of budget overrun, but the effect is not significantly different from zero. We enclose the results below in Table B1. We have chosen not to include these new results in paper, as the results are not significant. Although we are not with our current data able to credibly show harmful effects on child outcomes of budgetary concerns, we still think it is important to document that budget adherence may have unintended side-effects in the sense that child protection decisions are influenced by factors such as (within-year variation in) budget adherence, which is against the intention behind child protection laws and principles.*

3. A final remark on policy implications: the difference between day care and child protection is not just a matter of the structure of the costs (as suggested at p.26) but it is also a matter of the proportion of residents affected by the service. Using the descriptive stats, the authors should provide a flavor of how relevant are the population groups more likely to be interested by child protection in a given municipality in a given year. For instance, how relevant are single mums in a certain municipality, or immigrants? They could use these infos to run specific heterogeneities.

*Response:* *We acknowledge that our discussion was not clear and that the proportion who is affected by child protection also is important. We have expanded the discussion in the end of the Introduction and now explicitly discuss why child protection receives less political attention and why delegation to local levels might be problematic. We have also added an extra sentences in the Discussion and conclusion, mentioning the proportion of people affected as a reason for child protect being sensitive to budget concerns.*

*In terms of heterogeneity tests, we have performed a couple of new heterogeneity tests. These are documented in Tables A5 and A6 in the Appendix. We have now added estimations where we investigate for heterogeneous effects by splitting the sample according to fraction of citizens with primary school as the highest educational attainment, fraction of single parent, average income, more than 100 children in care in by 1^st^ January 2007. None of these factors cause significant heterogeneous effects.*

4. The measure of taking kids out of their home is an extreme ratio. There might be early intervention programs in order to avoid this extreme measure. Has anything happen in that respect during the observational period? It appears that there has been a decrease in the use of out-of-home procedure independent from the 2011 reform (see Figure A5), could it be due to the use of alternative solutions?

*Response: Thank you very much for this suggestion. We have added a column in table 1, where we analyze the number of children receiving preventive actions (such as mentoring programs and support for the family). We perform exactly the same analysis as for the number of children in out-of-home care. The results point in the same direction as the main analysis, and we find that the number of children receiving preventive action decreases if the budget share is high after 2011, whereas there is no effect before 2011.*

5. As for the empirical approach, why do not adopt a more simple scenario. Split municipalities using an aggregated measure referred to the period before the reform into more and less fiscal conservative (so to say, more or less available to run over expenditures). For instance using the average excess budget run in the pre-reform period. Then you know that the introduction of the 2011 reform will have a different intensity, and you can check the effect of the intensity of the treatment on child protection actions and expenditures. I find the use of the expenditures up to July 1st as an extra, rather than an intuitive baseline.

*Response: We have tried to follow this suggestion and calculated a measure of intensity using the pre-reform data. We use data on annual budget overrun defined as actual expenses divided by expenses in the budget for years 2007-2010 to construct the intensity of the reform. For each municipality, we calculate average budget overrun for the pre-reform. We then use the post reform data (2011-2016) and investigate the impact of the intensity on the number of children in care. We do find strong and negative effects of the intensity on the number of children in care (see the Table B2 below). Notice that in this analysis we only have variation in the intensity across municipalities and can therefore not control for municipality fixed effects. This is the main reason for why we have chosen not to show these results in the paper, as we suspect unobserved municipality factors such as the number of children at risk in the municipality that may be correlated with the intensity measure.* *Our current approach allows us to exploit both within and across municipality variation, whereas the suggested approach would leave us only with across municipality variation. We see a lot of variation in budget shares within municipalities across years and it is not clear that some municipalities are always high- or low-spending. For these reasons, we do not think that the suggested approach would yield any additional information. We have adjusted the text to underline that we exploit both within and across municipality variation.*

*Regarding the use of expenditures up to July 1st, we see this is as a way of illustrating the overall idea of the empirical analysis graphically. We have adjusted the text to make this clearer.*

6. As an extra heterogeneities I would consider the alignment between the local and the national government (i.e. more funding or more flexibility), and to consider the distance from the election month, which could affect spending decisions (conditional to the relevance of the affected population).

*Response: Thank you for these suggestions. We have run two additional heterogeneity checks. See Tables A5 and A6. First we define a dummy, which is one if the majority in the local council is from the same bloc as the national government (liberal/conservative versus social democrats). It turns out that being from the same bloc as the national government makes the municipality more sensitive to the budget. Second, we add a dummy for whether it is a local election year (2009 and 2013). It turns out that the effect of the budget share is stronger in election years after 2011 (which is in 2013).*

**Response to Reviewer #2**

Thank you for providing a number of constructive and insightful comments and suggestions. We have tried our best to respond to these in the response letter and to incorporate the suggestions in the paper to the extent possible. We think that the comments and suggestions received have significantly improved the revised version of the paper.

Below we address the comments and suggestions one by one. Our responses are marked by using indented blue font.

1. I do not have major comments as the paper seems technically sound. I do think it would potentially benefit from looking at other thresholds other than 60% of budget used halfway through the year. This could either be as robustness or to show a change in the effect by the amount of overrun. At a minimum, there needs to be some discussion about why the 60% threshold was used instead of something else.

*Response: The 60% threshold is used in the presentation of descriptive evidence in Figure 3. We have now checked that the overall picture conveyed in Figure 3 is indeed robust to the specific choice of threshold (see Figure B1, below).*

*Moreover, we would like to add that the main analysis is not based on any particular threshold, but we gather from your comments that this is not all that clear in the text. We have adjusted the text, in particular the section describing the empirical strategy, to better reflect the fact that our results do not depend on any particular threshold. Thank you for making us aware of this.*

2. Figures and tables need some additional notes in order to be self-contained. In particular, Figure 1, Table 1, and Figure 4 all need more explanation so that a reader could flip to them and understand them without referencing the text.

*Response: We have made additional notes for all the figures and tables in the text.*

3. The models in Table 1 need column numbers (1), (2), (3).

*Response: We have added column numbers to table 1.*

4. Authors refer multiple times to using a lag of the dependent variable in the regression. However, the dependent variable is change in log children, while the independent variable is log children, so it is not the lag of the dependent variable. This language needs to be adjusted to accurately reflect what is in the model. I am not as familiar with the term ADL model, but I think this term may not be accurate for the same reason.

*Response: We agree the text describing the error correction model was not clear and we have adjusted the text in section 3.2 on methods used in the municipal-level analysis and the text around table 1.*

5. I think it is worth mentioning that these results likely reflect a lower bound on the effect of budget constraints on placements since budget constraints are likely to affect decisions earlier in the year as well. They just become more salient and more of the uncertainty has been realized later in the year.

*Response: We agree. We have added the following sentence in section 5. Discussion and conclusion, end of paragraph 2: “The estimated effects on out-of-home care placements are likely to be a lower bound of the effects of budgetary restraint on decisions in the sense that budgetary constraints may impact decisions throughout the fiscal year.” (Included in line 469-471).*

6. I think Figure 2 would be more useful if it was shown as a ratio of the actual budget spent to the expected budget spent. An alternative may also be to add a dotted line for expected fraction of budget spent (assuming the same fraction is spent in each month). Either would make it easier to tell how far off the numbers are from their expected values.

*Response: Thank you for the suggestion. We have added the expected budget share to Figure 2.*

7. On page 12, when explaining the equation (2), it would be useful to explain who is included in each regression. So, the first one would include all children in care, presumably. The second one must include children at-risk for care by some measure, and I am not sure about the third.

*Response: We agree this was not clear from the text, and have added a section describing the samples more carefully. More detailed information can also be found in appendix table A2, which we now also reference in the text.*

8. I was surprised the interaction with post-2011 was not significant. Do the authors have any theories about that?

*Response: We do not find a significant effect of the post-2011 interaction in the municipality-level analysis on the total number of children in care reported in Table 1.*

*When looking more into the individual level data – shown in Table A4 in the appendix - we do however find significant effects of the post-2011 interaction for three sub-outcomes: the probability of ending a placement both before and after the age of 18, and the probability of moving to a cheaper placement. Thus, when looking at the combined effects of the main effect and the interaction effect in Figure A6 in the appendix, we see that the effects are not significant for these two outcomes before 2011, but they are significant after 2011. We see this as evidence that municipalities who were in danger of overrunning budgets looked for cuts in spending for the oldest of the children, and they looked for substitutions from more to less expensive treatments. We have strengthened the discussion of the interaction with 2011 in section 4.2*

9. On page 20, the authors say “The problem may be particularly relevant for small municipalities with fewer options for cost smoothing.” This is a testable hypothesis, so I would suggest that the authors test it in the data and report the findings.

*Response: Thank you for this comment. In the appendix, Table A5 and A6, we performed estimations where we interacted with a number of municipality characteristics. In general, the effects were not significantly different across these municipality types, except for a few outcomes, where political orientation, number of women, if the municipality has a sizable debt, if it is an election year and if the local council belongs political coalition as the prime minister. We briefly discuss the results in Section 4.4 of the paper. We have now added a short comment in Section 5, Discussion and conclusion, of “cost smoothing”.*

10. Compound adjectives need hyphens throughout the paper. For example: individual-level, low-spending, long-run, etc. all need hyphens when they are used as adjectives. Two-thirds and one-third also need hyphens.

*Response: Thank you. This has been corrected.*

11. Page 4, last sentence of the first paragraph: delete “which” and delete the apostrophe after citizens.

*Response: Thank you. This has been corrected.*

12. Early in the paper, it is mentioned which placements are more expensive and which less expensive. It would be helpful to provide a footnote later with this information for quick reference when it is mentioned again in the results (page 16, for example).

*Response: This information has been added to footnote 4.*

13. Page 17, line 353 should be “0.5 percentage point”

*Response: Thank you. This has been corrected.*

**Response to Reviewer #3**

Thank you for providing a number of constructive and insightful comments and suggestions. We have tried our best to respond to these in the response letter and to incorporate the suggestions in the paper to the extent possible. We think that the comments and suggestions received have significantly improved the revised version of the paper.

Below we address the comments and suggestions one by one. Our responses are marked by using indented blue font.

1. Although the authors place these results into a normative framework, ex ante it is not entirely clear whether spending more on various forms of child protection also brings adequate individual benefits. It is very easy to imagine narratives where this is the case, but it may support the normative statements better if the authors could give more background on the necessity and benefits of the different types of expenditures. E.g., how relevant or how much needed is out-of-home care for youth above 18 (which seems to be the most affected cohort by budgetary tightness); when this support is getting cut, is it a reasonable cost saving strategy or a government failure? The authors could help the readers to assess this by arguing about the relative benefits of different forms of care and types of expenditures more explicitly. All this may seem obvious, but stating it clearly would be helpful.

*Response: Thank you for raising this issue. We completely agree that it would strengthen our case if we could provide evidence on the potential harmful effects on children if child protection is influenced by budget concerns. We have been looking for outcomes for children where we could investigate the potential harmful consequences. Our challenge is that age of the children in our sample varies from 0-17 and it is difficult to find relevant outcomes. In our data, we have educational outcomes like completion of primary school, high school and vocational education. We have performed an analysis for a subsample of the children who are in out-of-home placement just before their 18th birthday as our results indicate that risk of budget overrun increases the likelihood of ending an out-of-home placement at 18. We investigate if the budget share affects the likelihood of completion of high school/vocational training by the age of 21. We do find evidence that after 2011 the probability of completion is lower for children in care in municipalities in risk of budget overrun, but the effect is not significantly different from zero. We enclose the results below in Table B1. We have chosen not to include these new results in paper, as the results are not significant. Although we are not with our current data able to credibly show harmful effects on child outcomes of budgetary concerns, we still think it is important to document that budget adherence may have unintended side-effects in the sense that child protection decisions are influenced by factors such as (within-year variation in) budget adherence, which is against the intention behind child protection laws and principles.*

2. Municipal-level strategy:
   a. Are budget shares based on an ex-post view so that they always amount to 1 by the end of the year? Or are they calculated in reference to the original municipal budgets, so that some municipalities may actually decide to overdraw (or undercut) their yearly budget on care at the costs (or benefits) of something else? Although this is described, I was still not sure which of the two is correct (providing basic descriptive statistics would have helped). If the former, does this affect the empirical strategy in any ways?

*Response: Thank you for pointing out that this was not clear enough in the text. We have clarified in the paragraph describing the budget share variable. The budget share exceeds one if a municipality has exceeded the planned annual budget. This should also be clear from Figure 2, which illustrates the variation in the budget share variable.*

b. Model 2 in table 1 shows insignificant interaction effects. But would this not invalidate the authors' argument that all these problems became more relevant after the new budgetary rules were introduced? At the beginning there are some arguments in this direction, but then there is virtually no discussion of whether this result invalidates the previous discussion.

*Response: We agree that we did not follow up on the discussion of the 2011 reform in the former version of the paper.*

*We do not find a significant effect of the post-2011 interaction in the municipality-level analysis on the total number of children in care reported in Table 1.*

*When looking more into the individual level data – shown in Table A4 in the appendix - we do however find significant effects of the post-2011 interaction for three sub-outcomes: the probability of ending a placement both before and after the age of 18, and the probability of moving to a cheaper placement. Thus, when looking at the combined effects of the main effect and the interaction effect in Figure A6 in the appendix, we see that the effects are not significant for these two outcomes before 2011, but they are significant after 2011. We see this as evidence that municipalities who were in danger of overrunning budgets looked for cuts in spending for the oldest of the children, and they looked for substitutions from more to less expensive treatments. We have strengthened the discussion of the interaction with 2011 in section 4.2.*

c. Column 3 of table 1: results are shown but not discussed. A non-attentive reader could miss entirely the meaning of this column, especially if only looking at the table.

*Response: We discuss the results of column 3 table 1 in Section 4.4 on Heterogeneous effects and robustness. However, we agree that since the table appears in the results section of the paper, it is natural to also comment on the result of the placebo test in the main results section. We have inserted a brief discussion of the interaction with 2011 in section 4.1, above Table 1 (line 348-350).*

3. Individual-level strategy:
   I had difficulties to clearly see the estimation strategy. Whereas the municipality-level regressions are fairly clear, I was somewhat confused by the individual-level estimates.
   a. First of all, I do not clearly see why are those estimates run at the individual level at all. The main variation is at the municipality-month level. Could not aggregate series be generated on the flows of children (number of below-18 ending placement, number of above-18 ending placement), etc.? I see that individual-level regressions offer the possibility to control for individual and household characteristics, but I doubt that these would change any of the results and many of the characteristics could also be accounted for by an individual fixed effect. Moreover, when looking at the individual-level estimations, it seems to me that some of these outcomes beg for a duration model, e.g. when referring to the likelihood of ending a spell. The individual-level results seem to be run at very different sample sizes, again raising the question whether sample selection would be relevant in any form here. Such concerns would be eliminated when looking at monthly flow data at the municipality level w/o losing any of the narrative content?

*Response: We agree that in principle, the analysis could be performed at the municipality level. However, in that case, we would have to weigh each municipality-level variable using a weight based on number of children in care or other weight. Moreover, we observe (Table A4) that individual-specific factors are important in decisions to place in out-of-home care. Thus, controlling for these individual factors strengthens our identification of municipal-level variation in budgets. Furthermore, we control for municipality-specific effects and cluster standard-errors at the municipal level.*

b. It is not clear to me why are year and month effects always listed differently. I would have thought that all models include simply time (which is here month-year) fixed effects. The way year and month dummies are separately emphasized, it seems like there are T year dummies, and 12 year-of-month dummies to account for seasonality. Instead, I would add Tx12 month FEs. Maybe this is what happening here, it could be then described as monthly FEs.

*Response: We add year dummies to take care of business cycle effects and structural changes unrelated to our main variables of interest. We add month dummies to account for seasonal variation. We use 11 month dummies and 9 year dummies. We have added a note to table 1, which makes this more explicit.*

c. From the individual-level regressions only selected marginal effects are plotted. As the results are not shown in the main paper, it is hard to imagine, how exactly these results are estimated (e.g., how the interaction effects of before 2011 and after enter the model)? I also did not understand why would these interaction effects refer to July (stated on line 348). In a monthly panel, the marginal effects average out municipalities with above 60% budget in any of the months?

*Response: We plotted the marginal effects in Figure 4 for July where we imagine municipalities might naturally take a half-year status of budgets, but they could be plotted for any other month or for an average across all months, as suggested. However, if we plotted the marginal effects across budget share for any month we would compare municipalities who had spent above 60 percent in April with municipalities who had spent above 60 percent in November. We think it is hard to interpret this average, which is why we have chosen to show the marginal effect at a specific month.*

4. Smaller comments
   a. The tables should clearly state the dependent variables used.

*Response: We agree that the tables in the paper were insufficient in describing the dependent variable. We have added notes to all tables and figures.*

b. The description of the estimating equation (2) would be clearer if it would include all nested fixed effects (and always in the same order): y_ikt, B_ik
   t-1, etc.

*Response: In the individual-level estimation, we include the same set of dummies (and in the same order) as in regression model for the municipality analysis (equation (2)): municipality dummies (96 dummies), month dummies (11) and year dummies (9). We hope that it is clearer in this version.*

c. There are a few typos: e.g., line 229.

*Response: Thank you for pointing this out.*

**Additional tables for response to the editor and reviewers**

**Table B1: Completion of secondary education at 21**

|  | (2) |
| --- | --- |
|  | Completed education at 21 |
|  | b/se |
| Girl | 0.080^***^ |
|  | 0.006 |
| Missing info, Mother | -0.155^***^ |
|  | 0.026 |
| Missing info, Father | 0.037^**^ |
|  | 0.014 |
| **Information mother** |  |
| Immigrant | 0.022^*^ |
|  | 0.010 |
| Descendant | -0.055 |
|  | 0.051 |
| Labor income | -0.023^*^ |
|  | 0.010 |
| Out of labor force | 0.036^***^ |
|  | 0.010 |
| Social Security | -0.033^**^ |
|  | 0.011 |
| Single parent | 0.020^**^ |
|  | 0.006 |
| Age | -0.006^***^ |
|  | 0.001 |
| Age squared | 0.000^***^ |
|  | 0.000 |
| Primary school | 0.002 |
|  | 0.017 |
| High school | -0.004 |
|  | 0.017 |
| Vocational training | 0.027 |
|  | 0.028 |
| Short further edu. | 0.026 |
|  | 0.038 |
| Intermediate further edu. | 0.029 |
|  | 0.019 |
| Long further edu. | 0.058^**^ |
|  | 0.021 |
| Unknown edu. | 0.000 |
|  | . |
| Budget share at 18 before 2011 | 0.005 |
|  | 0.017 |
| Budget share at 18 after 2011 | -0.018 |
|  | 0.018 |
| Constant | 0.059^*^ |
|  | 0.026 |
| Birth year dummies | Yes |
| Birth month dummies | Yes |
| Municipality dummies | Yes |
| N | 15,201 |

Notes: The sample consists of all children who are in out-of-home placement when they are 17.5 years old in the period 2007-2016. The dependent variable is a dummy variable for completion of secondary education (high school, vocational training etc.) at the age of 21. All explanatory variables are measured at the month prior to the 18^th^ birth day.

**Table B2: The number of children in out-of-home care**

| Dep. Var | Log(y) | ΔLog(y) |
| --- | --- | --- |
|  | b/se | b/se |
| Intensity | -0.651 | 0.000 |
|  | 0.934 | 0.003 |
| Log ($y_{it-1}$) |  | 0.000 |
|  |  | 0.001 |
| Constant | 5.444^***^ | -0.004 |
|  | 1.023 | 0.007 |
| Year dummies | Yes | Yes |
| Month dummies | Yes | Yes |
| N | 6984 | 6984 |
| $R^{2}$ | 0.024 | 0.012 |

Note: The variable y refers to the number of children in out of home care. The model is estimated using monthly data for the period 2011-2016 at the municipality level. The data set consists of 97 municipalities (The municipality of Copenhagen is excluded). In column (1) the outcome is log number children in care and in column (2) the outcome is changes in log number of children receiving preventive actions. The variable intensity is defined as average percentage budget overrun in the period 2007-2010. The estimations include 11 month dummies and 5 year dummies fixed effects. Standard errors in brackets are clustered at the municipality level.*, **, *** indicate significance at 0.05, 0.01 and 0.001 level.

**Figure B1: Descriptive figures with threshold at 50 percent**

Note: High (Low) spending municipalities in year *t* are defined as municipalities that in year *t* have spent more (less) than 50 percent of the annual budget by July 1^st^. Panel (a): Outcome is changes in log number of children in care. Panel (b) and (c): Outcome variable is monthly probability of ending an out-of-home placement for children below (above) 18. Panel (d): Outcome variable is monthly probability of initiating an out-of-home placement. Panel (e): Outcome is probability for a child (below 18) who is not currently receiving preventive actions and the left panel is for a child that receive preventive actions. Panel (f): Outcome is probability of choosing a “cheap” placement instead of an expensive placement. Confidence bands at 0.05 level. This figure is similar to figure 3 in the paper except the threshold for low spending is below 50 percent.
