# Supplementary material for: Trading off fiscal budget adherence and child protection
Source: PLoS One. 2022 Mar 24;17(3):e0261664. doi: 10.1371/journal.pone.0261664 (PMC8947134; doi:10.1371/journal.pone.0261664)
Supplement: Attachment — Submitted filename: Response letter_Revision 2.docx [file pone.0261664.s006.docx]

**Response letter, revision 2**

**PONE_D_21_06271**

**Reviewer #1**

*I appreciate the attempts of the authors to address all the raise concerns. Yet I do think they have to turn down the selling point on “We thus highlight that enforcing strict budget adherence can have unintended and potentially devastating side effects." They do show that there is a trade off and that there is a decrease in the outcome of interest, but they do not have a smoking gun on the cognitive performance or emotional response in the treated children. I would suggest only to be open to that in their narrative.*

Response: We have now downplayed this sentence so that we abstain from talking about possible effects on child outcomes. Instead, we write in the abstract, on the bottom of page 1, that:

“We thus highlight that enforcing strict budget adherence may be in conflict with social policy goals.”

**Reviewer #2**

*The authors have successfully addressed almost all of my concerns. I just think one minor change is necessary - when describing the individual-level analysis in Section 3.3, it needs to be clear that for 4) we are interested in the probability of receiving an out-of-home placement rather than preventive action (right now, it reads like it is comparing receiving either of these to receiving nothing). At the moment, that is only clear by looking in the Appendix. Thanks!*

Response: We have clarified this by changing the formulation in section 3.3 (bottom of page 14) to:

“4) initiating a preventive care measure rather than an out-of-home placement”.
